# Priming with intranasal lactobacilli prevents *Pseudomonas aeruginosa* acute pneumonia in mice

**DOI:** 10.1186/s12866-021-02254-7

**Published:** 2021-06-28

**Authors:** Marie-Sarah Fangous, Philippe Gosset, Nicolas Galakhoff, Stéphanie Gouriou, Charles-Antoine Guilloux, Christopher Payan, Sophie Vallet, Geneviève Héry-Arnaud, Rozenn Le Berre

**Affiliations:** 1 Laboratoire de biologie médicale, Centre Hospitalier de Cornouaille, Quimper, France; 2 Univ Brest, Inserm, EFS, UMR 1078, GGB, Brest, France; 3 University of Lille, CNRS UMR9017, Inserm U1019, CHRU Lille, Institut Pasteur de Lille, CIIL - Center for Infection and Immunity of Lille- OpInfIELD, Lille, France; 4 Département de Bactériologie-Virologie, Hygiène hospitalière et Parasitologie-Mycologie, CHRU La Cavale Blanche, Brest, France; 5 Département de Médecine Interne et Pneumologie, CHRU La Cavale Blanche, Brest, France

**Keywords:** *Lactobacillus*, Probiotics, Intranasal administration, *Pseudomonas aeruginosa*, Respiratory tract infection, Mice

## Abstract

**Background:**

Increasing resistance to antibiotics of *Pseudomonas aeruginosa* leads to therapeutic deadlock and alternative therapies are needed. We aimed to evaluate the effects of *Lactobacillus* clinical isolates in vivo*,* through intranasal administration on a murine model of *Pseudomonas aeruginosa* pneumonia.

**Results:**

We screened in vitro 50 pulmonary clinical isolates of *Lactobacillus* for their ability to decrease the synthesis of two QS dependent-virulence factors (elastase and pyocyanin) produced by *Pseudomonas aeruginosa* strain PAO1.

Two blends of three *Lactobacillus* isolates were then tested in vivo: one with highly effective anti-PAO1 virulence factors properties (blend named L.rff for *L. rhamnosus*, two *L. fermentum* strains), and the second with no properties (blend named L.psb, for *L. paracasei, L. salivarius* and *L. brevis*). Each blend was administered intranasally to mice 18 h prior to PAO1 pulmonary infection. Animal survival, bacterial loads, cytological analysis, and cytokines secretion in the lungs were evaluated at 6 or 24 h post infection with PAO1.

Intranasal priming with both lactobacilli blends significantly improved 7-day mice survival from 12% for the control PAO1 group to 71 and 100% for the two groups receiving L.rff and L.psb respectively. No mortality was observed for both control groups receiving either L.rff or L.psb. Additionally, the PAO1 lung clearance was significantly enhanced at 24 h. A 2-log and 4-log reduction was observed in the L.rff + PAO1 and L.psb + PAO1 groups respectively, compared to the control PAO1 group. Significant reductions in neutrophil recruitment and proinflammatory cytokine and chemokine secretion were observed after lactobacilli administration compared to saline solution, whereas IL-10 production was increased.

**Conclusions:**

These results demonstrate that intranasal priming with lactobacilli acts as a prophylaxis, and avoids fatal complications caused by *Pseudomonas aeruginosa* pneumonia in mice. These results were independent of in vitro anti-*Pseudomonas aeruginosa* activity on QS-dependent virulence factors. Further experiments are required to identify the immune mechanism before initiating clinical trials.

**Supplementary Information:**

The online version contains supplementary material available at 10.1186/s12866-021-02254-7.

## Background

Pulmonary infection with *Pseudomonas aeruginosa* in patients with cystic fibrosis (CF) and chronic obstructive pulmonary disease, is characterized by high morbidity and mortality [1, 2]. Recently, the prevalence of *P. aeruginosa* community-acquired pneumonia among those with chronic lung disease and already colonised with *P. aeruginosa* was 67% [3]. In parallel, antibiotic resistance is increasingly leading to therapeutic deadlock. The European Centre for Disease Prevention and control found that in 2019, 12.1% of *P. aeruginosa* invasive isolates were resistant to at least three main antimicrobial groups [4]. In the USA, multidrug resistant *P. aeruginosa* account for 32,600 cases of infection and 2700 deaths [5].

Many antibiotic alternatives, such as probiotics, have been tested. Probiotics are defined as living microorganisms which, when administered in adequate amounts, confer a health benefit to the host [6]. *Lactobacillus* spp., the most studied probiotic, can be isolated from food and human mucosa. Their beneficial effect operates through different means, especially immunomodulatory and antibacterial activities [6]. Exploring their interaction with *P. aeruginosa* is difficult and could not be evaluated through bactericidal activity. Indeed, lactobacilli produce acids that directly inhibited the growth of *P. aeruginosa* [7]. Few studies tried however to explore their antipathogenic abilities in vitro against *P. aeruginosa* and focused on their impact against *P. aeruginosa* virulence factors [7–9]. Several strains have been shown to harbour anti-elastase and anti-biofilm properties [7–9]. In vivo, only two studies evaluated on murine model of pneumonia the beneficial effect of lactobacilli administration against *P. aeruginosa* [10, 11]*.* Khailova et al. highlighted that oral administration of *L. rhamnosus* GG improves 7-day survival following *P. aeruginosa*-induced pneumonia. It is thought that regulatory T cells may play a role in this protection [11]. To date in man, randomized trials suggest that probiotics decrease the incidence of ventilator acquired pneumonia [12]; to counteract the many biases reported in these studies, a large randomized control trial is recruiting (PROSPECT study ClinicalTrials.gov Identifier: NCT02462590) [13].

While the oral route is often studied to analyse lactobacilli effects, the nasal route could provide benefits for respiratory infections by stimulating the activation of the lung immune response. Accordingly, studies show that intranasal administration of various *Lactobacillus* strains decrease mortality in viral pneumonia murine models [14, 15]. Recently, we conducted the first study that investigates the protective effect of intratracheally inoculated lactobacilli against *P. aeruginosa* acute pneumonia on a mouse model [16]. It highlighted that the respiratory route of *Lactobacillus* administration may prevent from *P. aeruginosa* infection by decreasing the bacterial lung load and modulating the cytokine levels. Lactobacilli used have been mainly isolated from diaries. We hypothesized that clinical lactobacilli strains could be more adapted to the lung, than other probiotics strains administrated by oral route and usually isolated from the gut or diaries.

In this study, in the light of our previous results, we’d like first to screen for new lung-adapted probiotic strains to consider for in vivo studies, and second to confirm the use of the respiratory route for their administration against *P. aeruginosa* lung infection*.* The screening involved *Lactobacillus* isolates that we previously obtained from CF expectorations [17]. It was based on their capacity to inhibit two *P. aeruginosa* QS-dependent virulence factors (elastase and pyocyanin). We then evaluated the effect of intranasal administration of two blends of three selected lactobacilli strains on *P. aeruginosa* murine acute pneumonia. Primary outcomes were the survival and bacterial lung load 24 h after *P. aeruginosa* induced pneumonia. To decipher the effects of lactobacilli, cytological analysis, chemokines, and cytokine secretions from bronchoalveolar lavage (BAL) were measured.

## Results

### In vitro screening of lactobacilli isolated from CF respiratory samples

Forty strains (80%) exhibited anti-elastolytic activity (mean activity = − 37.4% ± 0.15), and 12 (24%) exhibited anti-pyocyanin activity (mean activity = − 18.13% ± 0.15) (S1 Figure).

To constitute 2 blends of lactobacilli to administrate to the mice model of *P. aeruginosa* pneumonia, 3 strains with the highest anti-*P. aeruginosa* QS dependent virulence factor activities (L.rff) and 3 strains with no anti-*P. aeruginosa* QS dependent virulence factor activities (L.psb) were selected (S2 Table).

L.rff was constituted with 2 *L. fermentum* strains and 1 *L. rhamnosus strain* (*L. rhamnosus 2C, L. fermentum 9C* and *L. fermentum 10C*).

L.psb was constituted with 1 *L. paracasei,* 1 *L. salivarius* and 1 *L. brevis* strain (*L. paracasei 9 N, L. salivarius 20C* and *L. brevis 24C*).

### Nasal priming with lactobacilli enhances the survival rate

**Fig. 1:**
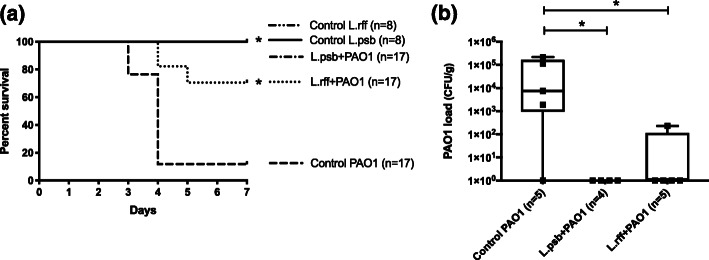
**a)** Mice survival rate*.* Priming of the respiratory tract with L.psb (1 × 10^6^ CFU/mouse) or L.rff (6 × 10^6^ CFU/mouse) resulted in survival in response to *P. aeruginosa* infection (6 × 10^6^ CFU/mouse). Statistical significance: *, *p* < 0.001 for the L.rff + PAO1 and L.psb + PAO1 groups compared to the control PAO1 group, and *p* < 0.05 for the L.rff + PAO1 group compared to L.psb + PAO1 group. **b)** Pulmonary *P. aeruginosa* burden measured on total lung homogenates. Priming of the respiratory tract with L.psb (2 × 10^5^ CFU/mouse) or L.rff (4 × 10^6^ CFU/mouse) 18 h prior to *P. aeruginosa* infection (6 × 10^6^ CFU/mouse) enhanced the clearance of *P. aeruginosa*. Statistical significance: *, *p* < 0.05 compared to the control PAO1 group

C57BL/6 mice were inoculated intranasally with each blend of lactobacilli 18 h prior to PAO1 administration (S3 Figure). All but two control PAO1 mice died (12% survival). Mice receiving L.rff responded with 71% survival (*p* < 0.001). Mice receiving L.psb were fully protected (100% survival) (*p <* 0.001 compared to PAO1 group and *p* < 0.05 compared to L.rff + PAO1 group). None of the control L.rff or control L.psb mice died or exhibited any clinical signs of distress (Fig. 1a).

### Administration of lactobacilli decreases the lung *P. aeruginosa* load

After 24 h a 2-log and 4-log reduction was observed in the L.rff + PAO1 and L.psb + PAO1 groups respectively, compared to the control PAO1 group (*p* < 0.001, Fig. 1b).

No increase in lactobacilli load was observed in either group. However, lactobacilli were still present in the lung 24 h after the instillation, with 1 × 10^4^ and 1 × 10^3^ CFU/g for the L.rff + PAO1 and L.psb + PAO1 groups respectively.

### White blood cell count and cytokine analysis in BAL fluid

**Fig. 2:**
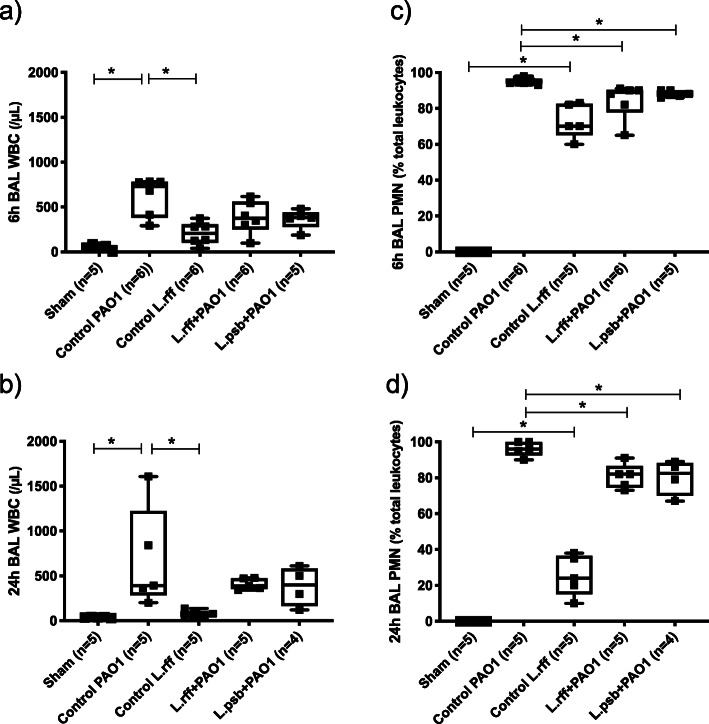
**a**) and **b**) Total white blood cell count in BAL fluids at 6 h and 24 h post infection with *P. aeruginosa*. **c**) and **d**) Polymorphonuclear cell ratio in BAL fluids at 6 h and 24 h post infection with *P. aeruginosa*. Statistical significance: *, *p <* 0.05; BAL, Bronchoalveolar lavage; PMN, Polymorphonuclear cells; WBC, White blood cells

To elucidate the mechanism of the *P. aeruginosa* lung load reduction, we investigated the white blood cell count (WBC) recruitment and cytokine synthesis in the BAL fluid (Fig. 2).

No difference was observed in the WBC at T6 and T24 between the control L.rff group and the control sham group. However, qualitatively, the BAL fluid infiltrate was mostly composed of polymorphonuclear (PMN) in the control L.rff group at T6 whereas the BAL fluid of sham mice only included alveolar macrophages (AM). As expected, mice from the control PAO1 group exhibited a strong increased number of WBC, which was mainly composed of neutrophils. This recruitment is more significant (3 and 9 times more respectively at T6 and T24) than in the control L.rff mice. A significant decrease of PMN in BAL fluid was observed at T6 and T24 in the L.rff + PAO1 and L.psb + PA01 groups compared to the PAO1 group.

We investigated the immunological response due to prophylactic administration of lactobacilli through pro-inflammatory cytokine and chemokine dosage in the BAL fluid. The anti-inflammatory cytokine IL-10 was also evaluated.

**Fig. 3:**
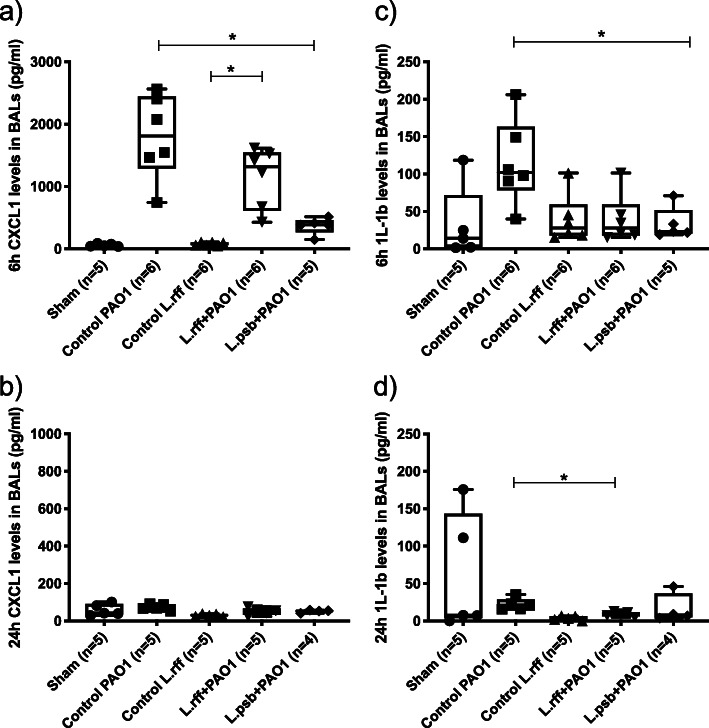
**a**) and **b**) CXCL1 levels in BALs at 6 h and 24 h post infection with *P. aeruginosa*. **c**) and **d**) IL-1b levels in BAL fluids at 6 h and 24 h post infection with *P. aeruginosa*. Statistical significance: *, *p <* 0.05; BAL, Bronchoalveolar lavage

**Fig. 4:**
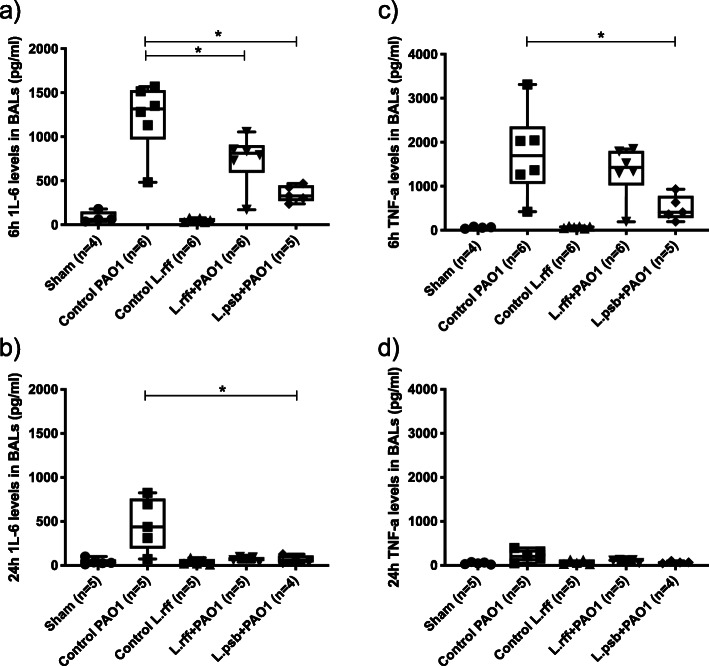
**a**) and **b**) IL-6 levels in BAL fluids at 6 h and 24 h post infection with *P. aeruginosa*. **c**) and **d**) TNF-α levels in BAL fluids at 6 h and 24 h post infection with *P. aeruginosa*. Statistical significance: *, *p <* 0.05; BAL, Bronchoalveolar lavage

**Fig. 5:**
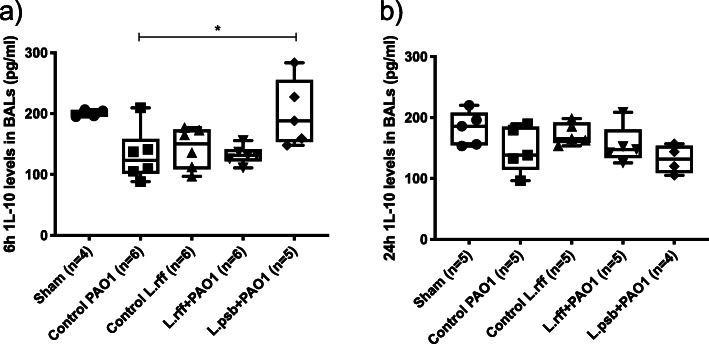
**a**) and **b**) IL-10 levels in BAL fluids at 6 h and 24 h post infection with *P. aeruginosa*. Statistical significance: *, *p* < 0.05. BAL, Bronchoalveolar lavage

Administration of lactobacilli alone did not induce the secretion of CXCL1, CXCL2, IL-1β, IL-6 and TNF-α compared to sham mice. Infection of PAO1 induced a cytokine burst particularly at 6 h. Prophylactic administration of lactobacilli leads to lower secretions of chemokines CXCL1 and CXCL2 (at T6) and proinflammatory cytokines IL-1β, IL-6 and TNF-α (both at T6 and T24) in both L.rff + PAO1 and L.psb + PA01 groups compared to the control PAO1 group (Figs. 3 and 4). Whereas PAO1 infection tends to decrease the IL-10 levels in the BAL, its production was significantly increased in the L.psb group compared to the *c*ontrol PAO1 groups (Fig. 5) but no difference was observed with the sham group.

## Discussion

In this study, intranasal administration of lactobacilli in a murine model of *P. aeruginosa* pneumonia significantly improved lung *P. aeruginosa* clearance 24 h post infection, and prevented mice death. This prophylactic treatment was associated with a lower secretion of chemokines and proinflammatory cytokines, and a lower neutrophil recruitment. This shows the implication of the innate immune response in this mechanism.

Pulmonary effects of *Lactobacillus* administration have been explored through oral gavage [10, 11, 18–22] or intranasal administration [14, 15, 23–26] and mainly focused on virus pathogens or *S. pneumoniae*. Oral gavage suggests that the protective abilities of *Lactobacillus* are based on the immunomodulation mediated by the gut-lung axis. Indeed, the gut microbiota interferes with the immune system, notably with granulocytosis by priming it through commensal components and pathogenic microorganisms which activate pattern recognition receptors (PRR) [27]. This reverberates on the lung and thus, any modification of the gut microbiota with oral administration of probiotics may influence lung immunity at distance. The lung microbiota may also directly interfere with the immune system through AM and dendritic cells which express various PRR. In our study, lactobacilli were administered intranasally in order to act directly on the airway mucosa immune system and to bypass the gut-lung axis (although swallowing cannot be totally excluded). We expected an enhanced, faster, and more appropriate response compared to the one following oral gavage. For the first time, our data showed that lactobacilli could sufficiently stimulate the respiratory mucosal immune system in situ to protect from *P. aeruginosa* infection. This approach already highlighted interesting results notably when used as a vaccine, against the flu or *S. pneumoniae* [28, 29]. This way of administration is otherwise safe for the mice, associated with a low number of PMN recruitment and absence of anti-*Lactobacillus* antibody secretion [24, 30, 31].

To our knowledge, our study is the first to explore the effects of intranasal administration of lactobacilli against *P. aeruginosa* respiratory infection. We observed an enhancement of lung *P. aeruginosa* clearance at 24 h post infection, associated with the decrease of chemokines and proinflammatory cytokines, the restoration of IL-10 and a reduction in neutrophil recruitment. The host immune response against acute *P. aeruginosa* infection is mainly based on the PMNs recruitment and activation, as neutropenic mice lead to higher *P. aeruginosa* mortality [32]. The PMNs recruitment is triggered secondly to *P. aeruginosa* interaction with AM or the airway epithelium, and through the toll like receptor signalling pathway [33]. However, an excess of PMNs was not always effective to clear the bacteria and caused serious tissue damage. A favourable outcome to *P. aeruginosa* infection is based on the adapted PMN recruitment, which allows for survival by clearance of the pathogen without excessive inflammation. The regulation of the inflammatory response is mainly based on anti-inflammatory cytokines, notably the IL-10 [34]. In our study, lactobacilli administered intranasally seemed to act on the innate immunity, particularly on PMNs. Indeed, PMNs were more significantly and transiently recruited in the control L.rff group compared to sham mice, with no consequence on the mortality, suggesting a beneficial preventive PMNs recruitment. After *P. aeruginosa* infection, the priming resulted in a reduced inflammatory response triggered by *P. aeruginosa*, and attenuated the recruitment of PMNs through a decreased secretion of proinflammatory cytokines and chemokines. Even with less PMNs, the *P. aeruginosa* clearance was enhanced, and the survival improved. Antibacterial effect of lactobacilli might be related to a priming of resident effector cells such as alveolar macrophages. Indeed, recent reports showed that lactobacillus strains are able to activate macrophages and to promote phagocytic and bactericidal activity in macrophages [35, 36]. Some previous studies focusing on priming with probiotics against various pathogens mostly agreed that probiotics could beneficially regulate the balance between pro and anti-inflammatory cytokines [18, 20, 25]. Others studies focusing on *P. aeruginosa* using the oral route to administer lactobacilli highlighted interesting results. According to Khailova and colleagues, the adaptive immunological response may be involved through regulatory T cells [11]. Alvarez and colleagues implicate the innate response, through an enhancement of the phagocytic activity of AM, associated with the activation of the specific response through secretion of IgA and IgM in the BAL fluid [10]. However, they didn’t observe any difference in white blood cell differential counts, confirming the interest of our work. In our study, we did not observe any lymphocyte recruitment following the administration of lactobacilli. However, we used different strains, means of administration, and mice. Our results may also implicate the innate response, through the modulation of the phagocytic activity conducted by decreased proinflammatory cytokines and chemokines, and increased IL-10. The *P. aeruginosa* clearance led by lactobacilli may be based on the modulation of the bactericidal and phagocytic activities of PMNs. The use of neutropenic mice may help to clarify the exact mechanism.

Lactobacilli have a long history of safe use by oral route [37] but the safety of lactobacilli application in the respiratory tract is crucial and needs to be studied. The longstanding dogma that the normal lung is free from bacteria [38] has been overturned by the recent advent of culture-independent techniques of microbial identification. Several publications showed that healthy human lungs contain diverse bacterial communities [39–41]. Additionally, in a recent study, Rosas-Salazar et al. [42] highlighted that lactobacilli was one of the 35 most abundant genus in the nasopharyngeal microbiome in infancy and showed a protective effect of upper airway colonization with Lactobacillus on asthma-related outcomes in children. Even with encouraging survival data, it will be necessary to analyse if the administration of *Lactobacillus* isolates in the respiratory tract lead to lung microbiota modifications, and to a particular microbiota signature already described in pulmonary diseases [43]. If *Lactobacillus* candidates are identified on the basis of convincing animal data, clinical trials phase I, II and III in humans will be essential to determine their safety.

*P. aeruginosa* pneumonia is still a lethal infection despite the use of antibiotics, especially among intensive care unit patients or patients with chronic respiratory diseases such as cystic fibrosis. There is an urgent need to develop alternative treatments to antibiotic therapy. Probiotics have demonstrated their capability to protect from pathogens through heterologous immunity. Several studies on the murine model of pneumonia highlighted that the administration of *Lactobacillus* could cross-protect from a later viral or bacterial infection [10, 11, 15, 18, 22, 23, 26]. Furthermore, their abilities to protect humans from pulmonary infections have also been demonstrated [6]. The heterologous immunity is thus expanding as a therapeutically strategy [44]. Various *Lactobacillus* strains are used to prevent respiratory infections, with probiotic activity being strain dependent [11, 45, 46]. Few authors tried to screen their own strains before experimenting [7, 21, 30, 31, 46]. Screening usually focuses on the strain’s ability to inhibit virulence factors of the studied pathogen [7, 46, 47], to produce inhibitory substances [30, 46] or cytokines [21], or to select the strains with the better adhesive properties in their experiment models [31, 46]. In our study, we screened for new lung-adapted probiotic strains with anti-*P. aeruginosa* abilities. This screening was based on a previous work, which studied the prevalence of lactobacilli in the lung of CF patients in regard to their *P. aeruginosa* colonisation status [17]. As no difference in species diversity or frequency was observed, we secondly screened a representative sample of 50 strains for their in vitro ability to inhibit two virulence factors produced by *P. aeruginosa,* the elastase and pyocyanine. These two major virulence factors are regulated by quorum sensing, cause a wide range of pathogenic effects and extensive tissue damage, and their pathogenicity has been confirmed in vivo on murine models of *P. aeruginosa* pneumonia [48, 49]. Furthermore, this screening strategy is based on the inhibition of two virulence factors after neutralising to pH 7 the coculture. This avoids the direct antibacterial effect due to *Lactobacillus* lactic and acetic acids production [7]. Our approach is original as we choose to highlight novel respiratory strains with great in vitro potential against *P. aeruginosa*. Despite thoughtful screening, improved survivals were observed whatever the blend of lactobacilli administered as a prophylactic treatment. Otherwise, the L.psb blend constituted with ineffective strains against *P. aeruginosa* QS-dependent virulence factors in vitro seemed to better act in vivo against *P. aeruginosa.* These results will deserve to be confirmed with other experiments but already raised a number of interesting questions concerning the mechanisms of action underlying the beneficial effect observed against *P. aeruginosa*. Additional are also required in order to determine the kinetic of the beneficial effect of our blends and their activity during chronic infection. The analysis of the lung clearance of our blends should be conducted in parallel. Although we report that administration of L.rff blend did not alter the health status in naïve mice and induce a mild and transient neutrophil recruitment not associated with a lung cytokine burst, these data should be extended.

A limitation of our study is related to the lack of data concerning the safety of the L.psb treatment about cytokine dosages and cytological analysis. However, it may be extrapolated to L.rff control group results. Indeed, in our first set of experiment, we focused on the prophylactic activity of the lactobacilli cocktail with a strong in vitro activity (L.rff cocktail) that we compared with a cocktail without in vitro activity (L.psb cocktail). Thus we only included a control group receiving the L.rff blend. Since the L.psb cocktail with no inhibitory abilities against *P. aeruginosa* had a similar in vivo activity against PAO1 during this first set of experiment, we added one supplementary L.psb control group receiving this blend for survival assay to evaluate their safety.

This study suggests that the lactobacilli abilities in vitro cannot predict their in vivo abilities to fight against *P. aeruginosa* pneumonia. The two QS-dependent virulence factors, elastase and pyocyanin, probably do not interfere directly with the immune response in our murine model of *P. aeruginosa* pneumonia. Indeed, numerous *P. aeruginosa*-virulence factors have been identified but the contribution of each of them in lung pathogenicity is not deciphered. In addition, further experiments to elucidate the precise mechanism of action are needed. Notably, the priming mediated by each single strain should be studied in distinct experimentations.

## Conclusions

In summary, we screened in vitro 50 *Lactobacillus* strains on their ability to inhibit the synthesis of 2 *P. aeruginosa* PAO1 virulence factors. Two blends of three *Lactobacillus* strains were constituted and intranasally administrated in a mice model of murine acute pneumonia. Our results firstly showed that intranasal administration of *Lactobacillus* strains can prevent from *P. aeruginosa* acute pneumonia by enhancing the mice survival, and modulating their local lung immunity. Secondly, in vitro abilities cannot predict lactobacilli in vivo abilities, as both blend of *Lactobacillus* improved mice survival, even when *Lactobacillus* strains administered didn’t diminished *P. aeruginosa* virulence factors production in vitro*.* The comprehension of the mechanisms involved in the immunomodulation requires further experimentation. The priming mediated by each single strain (alive or heat killed) of the promising L.psb cocktail (*L. paracasei* 9 N, *L. salivarius* 20C, *L. brevis* 24C) should be studied in distinct in vivo experimentations.

## Methods

### Inhibition tests of *Lactobacillus* strains on PAO1 on elastase and pyocyanin virulence factors

To administer *Lactobacillus* in the respiratory tract of mice, we screen for *Lactobacillus* isolates well adapted to the lung for their abilities to fight against *P. aeruginosa.* As lactobacilli produce acids that directly inhibit *P. aeruginosa,* bactericidal assays cannot be used for screening [7]. Fifty *Lactobacillus* strains previously isolated from respiratory samples from patients with CF [17] were thus screened in vitro for their ability to decrease the synthesis of 2 *P. aeruginosa* QS-dependent virulence factors, pyocyanin and elastase. *P. aeruginosa* PAO1 was chosen as the reference strain for all experiments [50]. Samples were stored at − 80 °C prior to subculturing on Mueller Hilton agar plates (MH) (bioMérieux) before experiments. All isolates were frozen at − 80 °C before subculture on 5% sheep-blood agar (bioMérieux) in 5% CO_2_ at 37 °C 2 days before experiments.

Elastase: PAO1 and each *Lactobacillus* isolate were separately cultivated overnight at 37 °C in Brain Heart Infusion broth (BHI) (Oxoïd) in 50 ml Falcon conical tubes. The inhibition of the elastolytic activity of PAO1 by *Lactobacillus* isolates was investigated by colorimetric assay, using Elastin Congo Red (ECR) (Sigma) as adapted by Alexandre et al. [7]. Succinctly, overnight aerobic culture of PAO1 in BHI broth under agitation was washed twice with isotonic saline solution and adjusted to 5 × 10^7^ CFU/ml in broth media. Overnight static culture of *Lactobacillus* in BHI broth was neutralised with NaOH 0,1 M to pH 7 and adjusted to 5 × 10^7^ CFU/ml in broth media. A 2 ml co-culture was made for each *Lactobacillus* isolates, by transferring 1 ml of the neutralised *Lactobacillus* broth to 1 ml of the PAO1 broth (vol/vol) and incubated in a tube under agitation 20 h under aerobic conditions at 37 °C. After centrifugation (20′ at 3500 g), 50 μl of the supernatant was mixed with 1 ml of Elastin Congo Red solution (20 mg/ml in a 10 mM sodium phosphate buffer) in a 2 ml-Eppendorf tube and incubated for 20 h more with rotation. Finally, the soluble fraction released in the supernatant by elastase was measured at 495 nm after centrifugation (20′ at 3500 g) in a microplate spectrophotometer (Multiskan FC Micro- plate Photometer, Thermo Scientific).

Pyocyanin: PAO1 was grown overnight in Bacto-Peptone (BP) broth (20 mg/l BP, MgCl_2_ 1,4 g/l, K_2_SO_4_ 10 g/l) (Oxoïd) and *Lactobacillus* isolates in MRS broth (bioMérieux). A 2 ml vol/vol co-culture was made as previously described for the elastase experiments, and incubate under aerobic conditions at 37 °C. The inhibition of the pyocyanin synthesis was investigated by colorimetric assay after extraction as previously described by Schaber et al. [51]. After centrifugation (20′ at 3500 g) of the co-culture, 50 μl of the supernatant was mixed with 50 μl of chloroform. The lower phase was transferred in a 15 ml Falcon conical tube and mixed with 2 ml of HCl (0.2 M). Finally, the pyocyanin extracted in the organic layer was measured at 520 nm in a microplate spectrophotometer (Multiskan FC Micro- plate Photometer, Thermo Scientific).

Pyocyanin and elastase results were normalised according to the OD_595_ of the co-culture and expressed as a ratio of the absorbance observed in presence of the *Lactobacillus* isolate to the absorbance observed with a monoculture of PAO1. Two experiments were conducted independently.

### Preparation of the bacterial strains

As commercial probiotics preparations are often made of several strains, a blend was constituted for the murine model of pneumonia. Three strains with the highest inhibitory abilities against PAO1 virulence factors were equally mixed in a blend named “L.rff*”* (composed of *L. rhamnosus 2C, L. fermentum 9C* and *L. fermentum 10C*). Three strains without inhibitory activity were mixed as a control in a blend named “L.psb” (*L. paracasei 9 N, L. salivarius 20C* and *L. brevis 24C*). Lactobacilli were grown overnight in MRS broth under aerobic conditions at 37 °C.

*P. aeruginosa* PAO1 was chosen as the reference strain for all experiments [50]. Samples were stored at − 80 °C prior to subculturing on Mueller Hilton agar plates (MH) (bioMérieux) before experiments. Then, PAO1 was grown overnight in Luria-Bertani broth (Sigma) under aerobic conditions at 37 °C. Each culture was washed twice with isotonic saline solution (SS) and adjusted to 10^9^ CFU/ml for the PAO1 suspension, or to 10^7^ CFU/ml for the L.rff and L.psb suspensions, based on the OD_595nm_ and controlled by serial dilution and plating on MH in triplicates.

### Murine model of acute pneumonia

This study (APAFIS#9623-2,017,040,717,237,994 and APAFIS#12025-2,017,110,311,134,961) has been approved by the french ethics committee for animal experiments n° 074 under the responsibility of the french ministry of higher education and research.

C57BL/6 J mice, aged 6-8 weeks old, were purchased from Janvier Labs (Le Genest Saint Isle, France) and maintained at the University of Brest, France. The mice were maintained in constant temperature (22 °C) and environment humidity room. Mice were fed ad libitum and monitored every eight hours until being sacrificed. All mice received human care.

For the cytokine dosages, cytological and bacterial burden experiments, mice were randomly assigned to 5 groups (*n* = 5 to 6): Sham mice, Control PAO1, Control L.rff and study group PAO1 + L.rff. A supplementary study group PAO1 + L.psb was add as a control of the PAO1 + L.rff group. The aim was to compare in vivo the effects conferred by the administration of three *Lactobacillus* strains with and without inhibitive properties against elastase and pyocyanin synthesis.

For the survival experiments, considering the results obtained from cytokine dosages, cytological and bacterial burden experiments, we add one more supplementary control *Lactobacillus* group named Control L.psb. Thus, 67 mice were randomly assigned to 5 groups (*n* = 8 for control L.rff and L.psb groups and *n* = 17 for Control PAO1, PAO1 + L.psb and PAO1 + L.rff groups).

The control mice (sham) received isotonic saline solution. Mice were intranasally inoculated with 20 μl of the bacterial suspension (10 μl per nostril), under intraperitoneal anaesthesia with ketamine/xylazine (100/10 mg/kg).

Lactobacilli suspension (L.rff or L.psb) was administered 18 h prior to infection with PAO1. The control PAO1 group received SS instead of lactobacilli. The L.rff and L.psb control groups received SS instead of PAO1.

Six (T6) or 24 h (T24) post infection with PAO1, mice were anesthetized with intraperitoneal injection of ketamine/xylazine (100/10 mg/kg) and sacrificed by intracardiac exsanguination. BAL was performed after euthanasia by cannulation of the trachea and injection and aspiration of 500 μl of SS three times.

Blood, BAL fluid, lung and spleen tissues were harvested from animals under aseptic conditions.

### Bacterial burden in lung homogenates

Mice were sacrificed at T24 and lungs removed and homogenized with SS with Ultra-Turrax. Bacterial loads of PAO1 and *Lactobacillus* blends were determined by plating serial dilutions of total lung homogenate on Cetrimide (bioMérieux) and MRS agar plates. Each dilution was plated in duplicate. Plates were incubated 24 h to 48 h at 37 °C under aerobic conditions. The limit of detection by platting was 40 CFU/ml. Colonies were identified using MALDI-TOF mass spectrometry (Microflex LT, Bruker Daltonics, Bremen, Germany).

### White blood cell count

The total white blood cell (WBC) count on BAL fluid was enumerated by a manual counting method with a hemocytometer (Kova slide) by light microscopy.

Alveolar macrophages (AM), polymorphonuclear cells (PMNs) and lymphocytes were differentiated after centrifugation, cytospins preparation and May-Grünwald-Giemsa staining.

### Cytokine measurement on BAL fluid

The cytokines studied were IL-1β, IL-6, IL-10, TNF-α, and the 2 chemokines CXCL-1 and CXCL-2. IL-1β, IL-6 and IL-10 (eBiosciences), TNF-α and the chemokines CXCL1 and CXCL2 (R&D System, Abingdon, UK) were determined in the BAL fluid by enzyme-linked immunosorbent assay (ELISA), using commercial kits according to the manufacturer’s recommendations. The lower levels of detection were 7 pg/ml for CXCL1 and CXCL2, 4 pg/ml for IL-1β and IL-6, 8 pg/ml for IL-10 and TNF-α.

### Survival experiments

Mice were monitored during 7 days after infection with PAO1. Fur aspect, activity, behavior, posture, eyelids, respiration, chest sounds, and body weight were followed frequently during the whole experiment, and scored from 1 to 4 according to the M-CASS scoring system [52]. When mice reached a total score of 11 during the day, buprenorphine was administered subcutaneously (0.05 mg/kg/12 h) for analgesia. Mice were sacrificed when they reached a score of 4 in the 8 parameters during the day, or in one parameter at night to prevent overnight death.

### Statistics

Results are presented as a boxplot. Comparisons between the groups were analysed by the Mann-Whitney test. The analysis of survival was performed with the Kaplan-Meier method. Results were considered statistically significant for *p* < 0.05. All statistical tests were performed using the R software.

## Supplementary Information

**Additional file 1: S1 Figure**. Anti-PA activities of the 50 *Lactobacillus* strains screened. **S2 Table**. Anti-PA activities of the 6 *Lactobacillus* strains selected to be administered to the mice. **S3 Figure**. Study design. **S4 Table**. ARRIVE Essential 10 checklist for animal research and Recommended Set of the National Centre for the Replacement Refinement and Reduction of Animals in Research.

## Data Availability

The datasets used and/or analysed during the current study are available from the corresponding author on reasonable request.

## Abbreviations

AM: Alveolar macrophages
BAL: Bronchoalveolar lavage
BHI: Brain heart infusion
CF: Cystic fibrosis
CFU: Colony forming unit
MH: Mueller Hilton agar plate
OD: Optical density
PRR: Pattern recognition receptors
PMN: Polymorphonuclear
SS: Isotonic saline solution
