# Supplementary material for: Priming with intranasal lactobacilli prevents Pseudomonas aeruginosa acute pneumonia in mice
Source: BMC Microbiol. 2021 Jun 28;21:195. doi: 10.1186/s12866-021-02254-7 (PMC8237558; doi:10.1186/s12866-021-02254-7)
Supplement: Supplementary file 1 — Additional file 1: S1 Figure. Anti-PA activities of the 50 Lactobacillus strains screened. S2 Table. Anti-PA activities of the 6 Lactobacillus strains selected to be administered to the mice. S3 Figure. Study design. S4 Table. ARRIVE Essential 10 checklist for animal research and Recommended Set of the National Centre for the Replacement Refinement and Reduction of Animals in Research. [file 12866_2021_2254_MOESM1_ESM.docx]

**S1 Figure. Anti-PA activities of the 50 *Lactobacillus* strains screened**. Results are expressed as a ratio of the absorbance observed in the co-culture PAO1/*Lactobacillus* to the absorbance observed with a monoculture of PAO1, related to 1: Inhibitive activity = 1-[OD co-culture PAO1/*Lactobacillus*]/[OD monoculture PAO1]. Thus, the inhibitive activity of a monoculture of PAO1 is 0. When results are positive, inhibitive properties are observed against the virulence factors. When results are negative, no inhibitive properties are observed. The higher the percentage is, the stronger is the inhibitive property observed.

*, Selected strains with the highest anti-PA activity (L.rff blend); #, Selected strains with the weakest anti-PA activity (L.psb blend).

**S2 Table. Anti-PA activities of the 6 *Lactobacillus* strains selected to be administered to the mice**. Results are expressed as a ratio of the absorbance observed in the co-culture PAO1/*Lactobacillus* to the absorbance observed with a monoculture of PAO1, related to 1: Inhibitive activity = 1-[OD co-culture PAO1/*Lactobacillus*]/[OD monoculture PAO1]. Thus, the inhibitive activity of a monoculture of PAO1 is 0. When results are positive, inhibitive properties are observed against the virulence factors. When results are negative, no inhibitive properties are observed. The higher the percentage is, the stronger is the inhibitive property observed.

| Blend of lactobacilli | Strains | Anti-elastolytic activity | Anti-pyocyanin synthesis |
| --- | --- | --- | --- |
| “L.rff” | *L. rhamnosus* 2C | 0,39 | 0,29 |
|  | *L. fermentum* 9C | 0,51 | 0,06 |
|  | *L. fermentum* 10C | 0,31 | 0,24 |
| “L.psb” | *L. paracasei* 9N | -0,87 | -0,50 |
|  | *L. salivarius* 20C | -0,42 | -0,74 |
|  | *L. brevis* 24C | -0,15 | -0,16 |

**S3 Figure. Study design.** C57BL/6 mice were intranasally inoculated with lactobacilli (either L.rff or L.psb blend) or SS 18 h prior intranasally infection with PAO1.
SS, isotonic saline solution


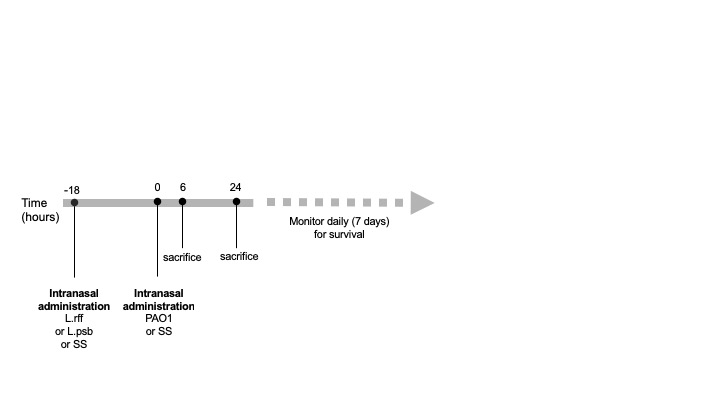


**S4 Table. ARRIVE Essential 10 checklist for animal research and Recommended Set of the National Centre for the Replacement Refinement and Reduction of Animals in Research.**

| ARRIVE essential 10 | | | |
| --- | --- | --- | --- |
|  |  | Recommendation | Section/  paragraph |
| Study design | 1 | For each experiment, provide brief details of study design including:   1. The groups being compared, including control groups. If no control group has been used, the rationale should be stated. 2. The experimental unit | Methods, paragraph 3 |
| Sample size | 2 | a. Specify the exact number of experimental units allocated to each group, and the total number in each experiment. Also indicate the total number of animals used.  b. Explain how the sample size was decided. Provide details of any a priori sample size calculation, if done. | Methods paragraph 3 |
| Inclusion and exclusion criteria | 3 | a.Describe any criteria used for including and excluding animals during the experiment, and data points during the analysis. Specify if these criteria were established a priori. If no criteria were set, state this explicitly.  b.For each experimental group, report any animals, experimental units, or data points not included in the analysis and explain why. If there were no exclusions, state so.  c.For each analysis, report the exact value of n in each experimental group. | Methods paragraph 3 and 4 |
| Randomisation | 4 | a.State whether randomisation was used to allocate experimental units to control and treatment groups. If done, provide the method used to generate the randomisation sequence  b.Describe the strategy used to minimise potential confounders such as the orders of treatments and measurements, or animal/cage location. If confounders were not controlled, state this explicity. | Methods paragraph 3 |
| Blinding | 5 | Describe who was aware of the group allocation at the different stages of the experiment | Methods paragraph 3 |
| Outcome measures | 6 | a.Clearly define all outcome measures assessed  b.For hypothesis-testing studies, specify the primary outcome measure, ie, the outcome measure that was used to determine the sample size. | Methods paragraph 3 and 4  Statistics |
| Statistical methods | 7 | a.Provide details of the statistical methods used for each analysis, including software used.  b.Describe any methods used to assess whether the data met the assumptions of the statistical approach, and what was done if the assumptions were not met. | Statistics |
| Experimental animals | 8 | a.Provide species-appropriate details of the animals used, including species, strain, and substrain, sex, age or developmental stage, and, if relevant, weight.  b.Provide further relevant information on the provenance of animals, health/immune status, genetic modification status | Methods paragraph 3 |
| Experimental procedures | 9 | For each experimental groups, including controls, describe the procedures in enough details to allow others to replicate them | Methods paragraph 2, 3, 4, 5, 6 and 7 |
| Results | 10 | For each experiment conducted, including independent replications, report :  a.summary/descriptive statistics for each experimental group, with a measure of variability where applicable.  b.If applicable, the effect size with a confidence interval | Results and statistics |
| ARRIVE recommended set | | | |
| Abstract | 11 | Provide an accurate summary of the research objectives, animal species, strain and sex, key methods, principal findings, and study conclusions | Abstract |
| Background | 12 | a.Include sufficient background to understand the rationale and context for the study, and explain the experimental approach.  b.Explain how the animal species and model used address the scientific objectives and, where appropriate, the relevance to human biology | Background |
| Objectives | 13 | Clearly describe the research question, research objectives and, where appropriate, specific hypotheses being tested. | Background paragraph 3 |
| Ethical statement | 14 | Provide the name of the ethical review committee or equivalent that has approved the use of animals in this study, and any relevant licence or protocol numbers. If ethical approval was not sought or granted, provide a justification. | Ethical approval and consent to participate |
| Housing and husbandry | 15 | Provide details of housing and husbandry conditions, including any environnmental enrichment. | Methods paragraph 3 |
| Animal care and monitoring | 16 | a.Interpreted the results, taking into account the study objectives and hypotheses, current theory, and other relevant studies in the literature.  b.Comment on the study limitations, including potential sources of bias, limitations of the animal model, and imprecision associate with the results. | Methods paragraph 3 |
| Interpretation/scientific implications | 17 | a.Interpret the results, taking into account the study objectives and hypotheses, current theory, and other relevant studies in the literature.  b.Comment on the study limitations, including potential sources of bias, limitations of the animal model, and imprecision associated with the results. | Discussion |
| Generalisability/translation | 18 | Comment on whether, and how, the findings of this study are likely to generalise to other species or experimental conditions, including any relevance to human biology. | Discussion |
| Protocol registration | 19 | Provide a statement indicating whether a protocol was prepared before the study, and if and where this protocol was registered. | Ethics approval and consent to participate |
| Data access | 20 | Provide a statement describing if and where study date are available. | Availability of data and materials |
| Declaration of interests | 21 | a.Declare any potential conflicts of interest, including financial and nonfinancial. If none exist, this should be stated.  b.List of all funding sources and the role of the funder(s) in the design, analysis, and reporting of the study. | Competing interest |
